# Clinical observation of a novel technique: transscleral suture fixation of a foldable 3-looped haptics one-piece posterior chamber intraocular lens implantation through scleral pockets with intact conjunctiva

**DOI:** 10.1186/s12886-019-1113-2

**Published:** 2019-05-09

**Authors:** Shuang Ni, Wei Wang, Xiang Chen, Xingdi Wu, Suhong He, Yajuan Ma, Wen Xu

**Affiliations:** 1 Eye Center of the Second Affiliated Hospital, Zhejiang University School of Medicine, Hangzhou, China; 2 Suichang Hospital of Traditional Chinese Medicine, Suichang, China; 3 Zhejiang Rongjun Hospital, Jiaxing, China

**Keywords:** Absence of capsule support, Posterior chamber intraocular lens, Scleral pocket, Transscleral suture fixation

## Abstract

**Background:**

To present the follow-up outcomes of a modified technique of transscleral suture fixation of posterior chamber intraocular lens (PCIOL) in eyes with inadequate capsule support.

**Methods:**

A retrospective chart review of 21 patients underwent transscleral suture fixation of a foldable 3-looped haptics one-piece PCIOL implantation through scleral pockets was conducted. Preoperative data and follow-up data for at least 3 months were collected for all patients.

**Results:**

The mean operative duration was 36.62 ± 10.70 min. The mean pre- and post-operative LogMAR uncorrected distance visual acuity was (1.25 ± 0.50 vs. 0.41 ± 0.22, *P* < 0.01). The mean pre- and post-operative LogMAR best corrected visual acuity was (0.48 ± 0.25 vs. 0.33 ± 0.24, *P* < 0.01). The mean proportion of postoperative endothelial cell loss was 11.46 ± 4.78%. The mean postoperative anterior chamber depth was 3.05 ± 0.44 mm. The mean postoperative IOL tilt degree was 2.81 ± 1.41^°^, and the mean postoperative IOL decentration degree was 0.31 ± 0.13 mm. Four patients with transient corneal edema (19.0%) and three patients with transiently elevated IOP (14.3%) were observed after operation, and such complications were resolved within 1 week. No severe complications were observed.

**Conclusions:**

The modified technique was a feasible method of PCIOL implantation.

**Electronic supplementary material:**

The online version of this article (10.1186/s12886-019-1113-2) contains supplementary material, which is available to authorized users.

## Background

Modern cataract surgery requires an adequate capsular support to implant the posterior chamber intraocular lens (PCIOL) inside the capsular bag [1]. However, certain situations, such as congenital and acquired zonular weakness, post-traumatic subluxation, post-lensectomy and post-complicated cataract surgery, would make the capsular-implantation of PCIOL impossible [2]. Under such conditions, aphakia can be effectively corrected by several surgical techniques, including anterior chamber angle IOL (ACIOL) and iris-fixated IOL, as well as scleral fixation of PCIOL [3]. Compared with other methods, scleral fixation of PCIOL has several inherent advantages, such as locating PCIOL in a position closest to the original lens, keeping PCIOL far from corneal endothelium and trabecular meshwork, and making PCIOL act as a mechanical barrier between vitreous cavity and anterior chamber [4]. Furthermore, this method is also suitable for cases of severe iris trauma [5].

Since first reported by Malbran et al., transscleral suture fixation of PCIOL has become one of the most effective scleral fixation methods [6]. Various modifications of this technique have been reported [7]. However, due to the complexity of this technique, the long surgical duration may increase the risk of complications, such as hyphema, vitreous hemorrhage or cystoid macular edema (CME) [7]. On the other hand, the method for PCIOL fixation in an accurate position needs to be improved, as only half of the PCIOLs are seen in the intended position after transcleral suture fixation of PCIOL [8]. Suture degradation and subsequent progressive loosening of the PCIOLs’ haptic can induce optic disc tilting in sutured scleral-fixated PCIOL [9]. Serious PCIOL tilt or decentration will result in oblique astigmatism and can also cause myopic shift and lateral shift of focus [7, 10]. In addition, conjunctival dissection required in almost every surgical technique may cause postoperative ocular surface scarring, which not only affects patient comfort, but also increases the difficulty of possible future glaucoma surgery [4]. In order to attenuate the above-mentioned potential risks, we modified the surgical technique to avoid conjunctival dissection by making three scleral pockets and implanting a foldable 3-looped haptics one-piece PCIOL using a stable 6-point transscleral suture fixation. Moreover, we also described the operative technique and estimate its anatomic, visual and safety outcomes.

## Methods

### Patients and assessment

A retrospective chart review consisting of 21 patients who underwent modified transscleral sutured PCIOL implantation between January 2017 and August 2018 was performed. The inclusion criteria were as follows: patients who underwent transscleral suture fixation of PCIOL; time between the primary surgeries and transscleral sutured PCIOL implantation surgery was at least 3 months; the retina remained attached preoperatively; no preoperative active intraocular inflammation; the preoperative intraocular pressure (IOP) was < 25 mmHg; and the preoperative endothelial cell count (ECC) was > 1200 cells/mm^2^. All the surgeries were performed by the same surgeon (W.X.). The information, including demographic data, ocular history, operative duration, preoperative and postoperative uncorrected distance visual acuity (UDVA), best corrected visual acuity (BCVA), IOP and ECC, postoperative anterior chamber depth (ACD), PCIOL tilt degree, PCIOL decentration degree, and postoperative complications, was collected. The duration of data collection for follow-up period was longer than 3 months after surgery for all patients.

### Surgical technique

The surgical steps were as follows: **(***see Video,* Additional file 1**)**

**Fig. 1:**
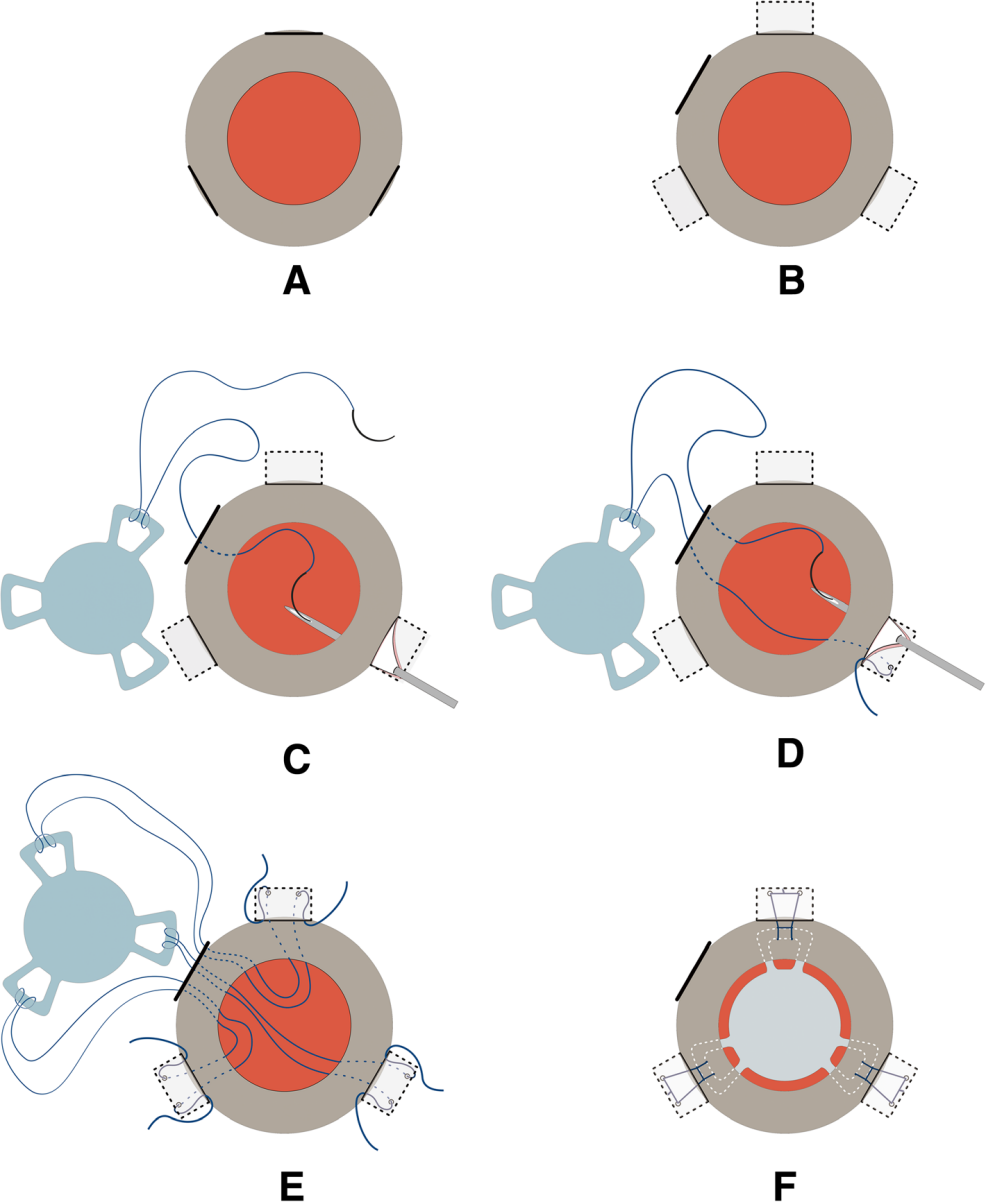
Schematic procedures of modified technique for PCIOL fixation. **a** Three 3-mm wide limbus-parallel half-thickness corneal incisions were made at the limbus. **b** Three 3 × 2 mm scleral pockets were dissected. A 3.5-mm wide superior corneal incision was made. **c** A double-armed suture was tied to one haptic of the PCIOL, and one of its needle suture was passed through the superior corneal incision and guided out of the eye from scleral pockets by a docking hollow needle. **d** The other needle suture was passed through the superior corneal incision and guided out at an adjacent position in the same way. **e** Sutures were tied on every haptic of the PCIOL and guided out of the eye from their corresponding scleral pocket beds. **f** The PCIOL position was set by adjusting tension of each suture knot

**Fig. 2:**
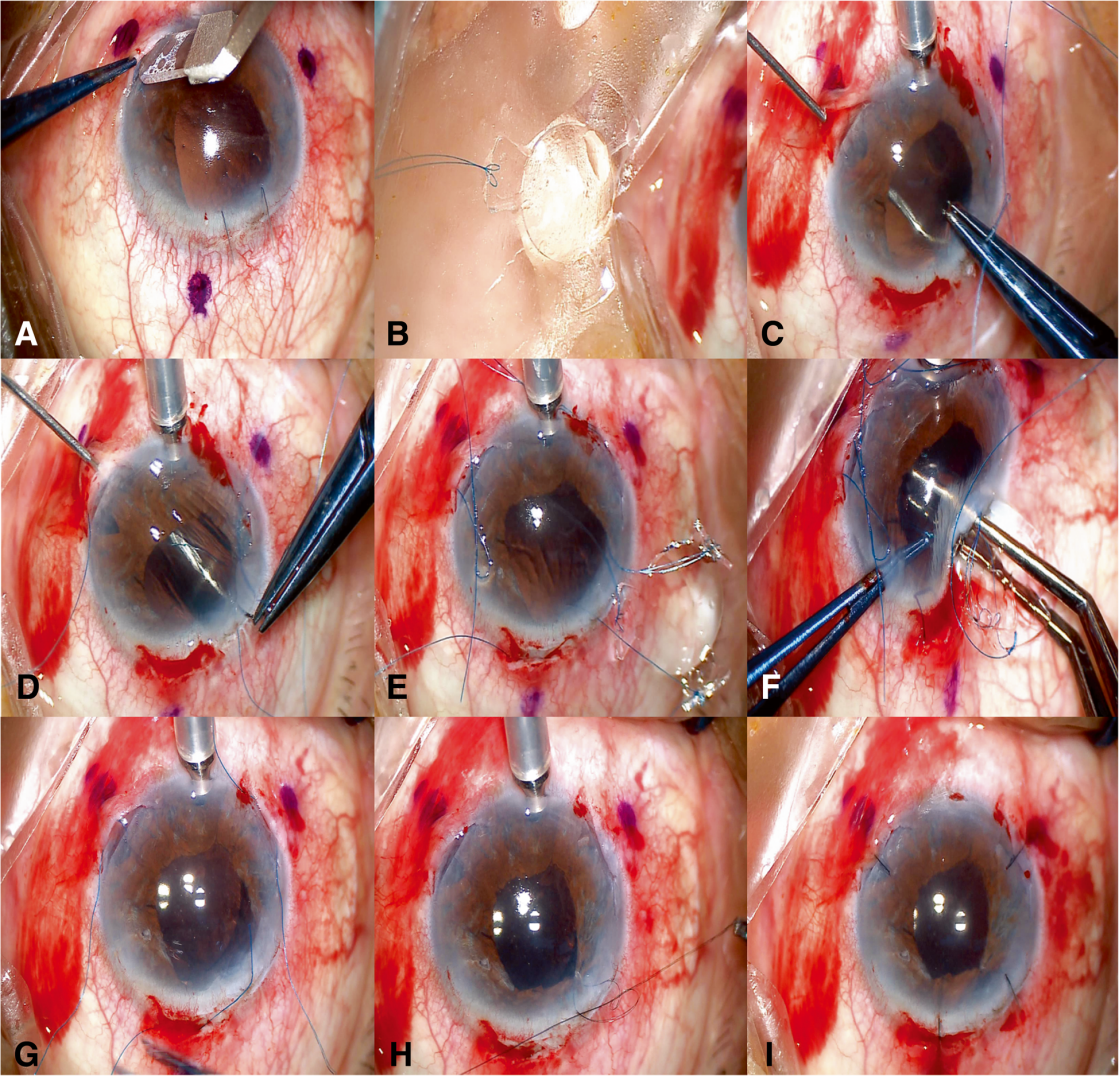
Operative photos of the modified technique for PCIOL fixation. **a** Three scleral pockets were made at marked positions. **b** A double-armed suture was tied to one haptic of the PCIOL by cow-hitch knot. **c** A hollow needle was passed through the scleral pocket bed into the eye to dock and guide out the suture needle coming from the superior corneal incision. **d** Another hollow needle was inserted into the eye at an adjacent point to dock and guide out the other needle of the previous double-armed suture. **e** Sutures were tied on every haptic of the PCIOL and guided out of the eye from their corresponding scleral pocket bed. **f** The PCIOL was folded and inserted through the superior incision. **g** The PCIOL position was set by adjusting tension of each suture knot. **h** Severe iris and pupil damage was repaired. **i** Scleral pockets and superior corneal incision were sutured

1. A retrobulbar block was recommended with a 1:1 mixture of 2% lidocaine and 0.75% bupivacaine to allow for adequate akinesia was recommended.
2. A 20-G infusion cannula was inserted at the limbus at about 6 o’clock to maintain the IOP for prevention of intraoperative globe collapse.
3. Three marks were made at conjunctiva 2 mm posterior to the limbus at 4, 8 and 12 o’clock by a maker pen. A 3-mm wide limbus-parallel half-thickness corneal incision was made at the limbus according to each marked position (Fig. 1a). Then three 3 × 2 mm scleral pockets were posteriorly dissected using a crescent blade (Figs. 2a and 1b).
4. A 3.5-mm wide superior corneal incision was made at 10 o’clock for anterior vitrectomy and PCIOL insert (Fig. 1b). A 1.0-mm paracentesis was created from clear corneal at about 2 o’clock as an auxiliary incision.
5. All of the lenticular tissues, including nuclear fragments, cortex, capsular remnants and dislocated IOL (if present), as well as vitreous in the anterior chamber and pupillary region, were removed. The anterior chamber was filled with a cohesive ophthalmic viscosurgical device (OVD).
6. A 3-looped haptics one-piece foldable PCIOL (CT ASPHINA 603P, Zeiss) was used, and the PCIOL power was reduced 1D from the predicted in-the-bag refraction.
7. A double-armed 8–0 polypropylene suture (PROLENE* 8752, Ethicon) was tightly tied to one haptic of the PCIOL by cow-hitch knot (Figs. 1d and 2b).
8. A 27-G hollow needle was used to scoop up the roof of one scleral pocket, vertically penetrated through pocket bed at its bottom about 2 mm posterior to the surgical limbus and inserted into the eye, and then it was passed behind the iris until its tip appeared in the center of the pupil. A suture needle was passed through the superior corneal incision, docked into the hollow needle and then guided through the scleral pocket bed out of the eye from the pocket (Figs. 1c and 2c).
9. A 27-G hollow needle was inserted into the eye at an adjacent point and also 2 mm posterior to the surgical limbus on the same scleral pocket bed. The other needle of the previous double-armed suture tied on the same haptic was guided out in the same way (Figs. 1d and 2d).
10. The steps 7 to 9 mentioned above were repeated at the other two scleral pockets, respectively (Figs. 1e and 2e).
11. The PCIOL was folded, then inserted through the superior incision and placed in the ciliary sulcus (Fig. 2f).
12. The pairs of sutures at each pocket were knotted via tension adjustable knot to center the optic of the PCIOL. After the PCIOL was placed in the exact position and each knot was placed into scleral pocket, and the suture ends were laid flat into the scleral pockets respectively (Fig. 1f and 2g).
13. Severe iris and pupil damage was repaired by 10–0 suture (Fig. 2h). The OVD was removed by irrigation and aspiration system, and then the infusion cannula was removed.
14. Scleral pockets and superior corneal incision were sutured by 10–0 suture, and knots were rotated into the eye (Fig. 2i). The 10–0 suture would be removed at 4 weeks after operation.

Postoperatively, topical antibiotic, steroidal anti-inflammatory and non-steroidal anti-inflammatory drops were given four times a day for 4 weeks.


**Additional file 1:** Modified transscleral suture fixation of PC-IOL.mp4. (MP4 19968 kb)


### Statistical analysis

Data were entered in a Microsoft Excel and were analyzed using SPSS version 16. Continuous variables were expressed as mean values ± standard deviations, and categorical variables were expressed as individual counts. Data were analyzed using *t*-test. Differences were considered statistically significant at *P* < 0.05 for all tests.

## Results

Table 1 summarizes the patient information. This technique was performed in 21 patients (15 males and 6 females). The mean age of the patients was 54.7 ± 16.8 years old, ranging from 14 to 70 years. The mean follow-up time was 7.8 ± 4.0 months, ranging from 3 to 20 months. The indications for surgery were aphakia, subluxated PCIOL, subluxated crystalline lens and exchange of ACIOL. A total of 11 patients had history of serious ocular trauma. Five patients underwent pars plana vitrectomy.

**Table 1:** Patient information

Parameter	
No. of eyes (*n* of pts)	21 (21)
Male gender, *n* (%)	15 (71.4)
Mean age (y)	54.7 ± 16.8
Range (y)	14–70
Mean follow-up time (m)	7.8 ± 4.0
Range (m)	3–20
Cause of transscleral PCIOL fixation, *n* (%)	
Aphakia	8 (38.1)
Subluxated PCIOL	5 (23.8)
Subluxated crystalline lens	7 (33.3)
Exchange of ACIOL	1 (4.8)
History of serious ocular trauma, *n* (%)	11 (52.4)
History of pars plana vitrectomy, *n* (%)	5 (23.8)

*PCIOL* posterior chamber intraocular lens, *ACIOL* anterior chamber intraocular lens

**Fig. 3:**
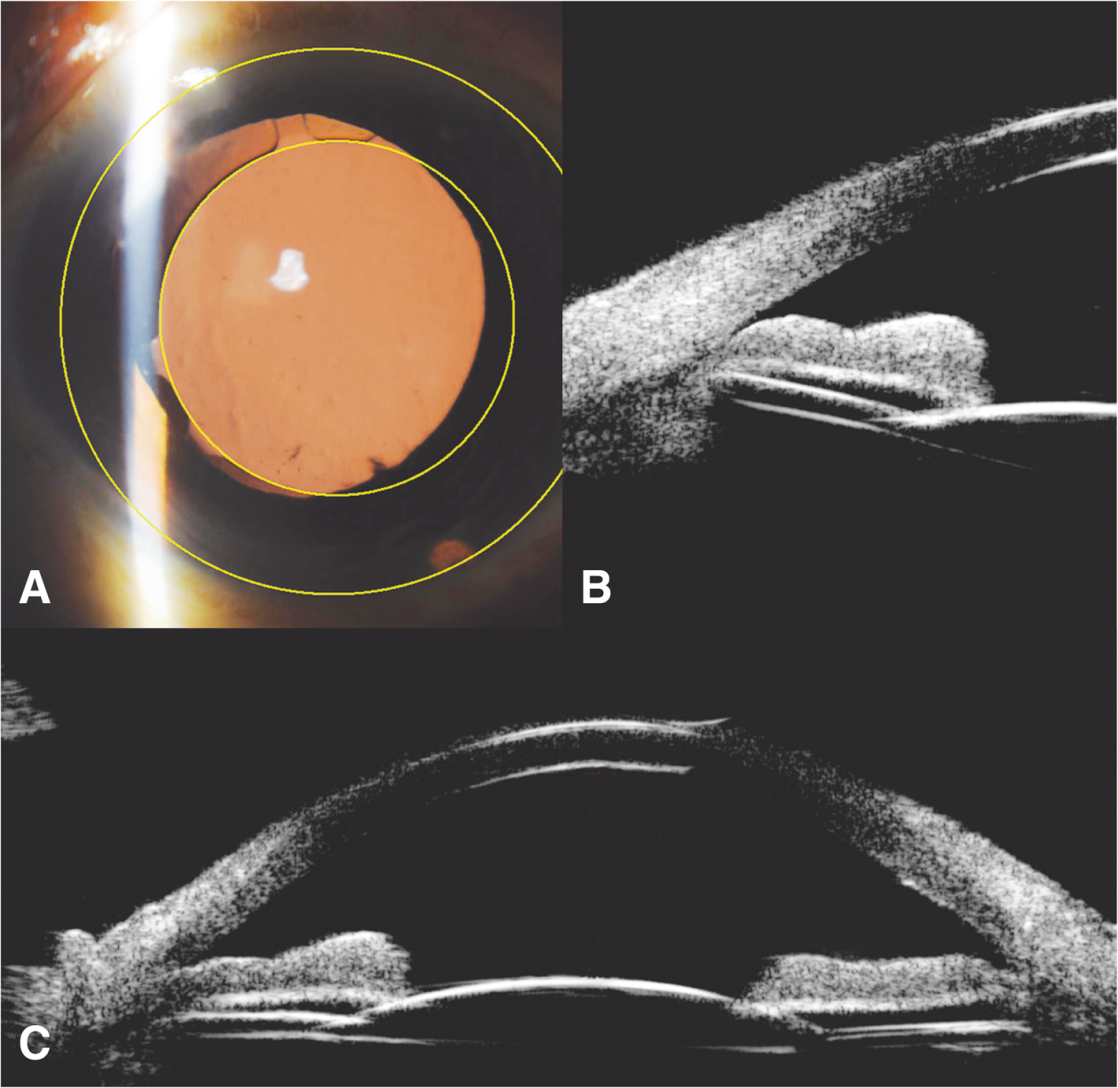
Anterior segment and UBM images of the implanted PCIOL. **a** Anterior segment photography of the PCIOL. The yellow circles represented the edge of PCIOL optic and best-fit circles created from upper and lower limbus. **b** UBM image showed that the haptic of PCIOL was placed at ciliary sulcus. **c** A typical UBM imaging with a PCIOL implanted

Table 2 lists the functional results. The mean operative duration was 36.62 ± 10.70 min. The mean postoperative LogMAR UDVA was better than the preoperative LogMAR UDVA (0.41 ± 0.22 versus 1.25 ± 0.50, *P* < 0.01). The mean postoperative LogMAR BCVA was better than the preoperative LogMAR BCVA (0.33 ± 0.24 versus 0.48 ± 0.25, *P* < 0.01). There was no significant difference between postoperative and preoperative IOP (14.13 ± 3.97 versus 13.42 ± 3.51 mmHg, *P* > 0.05). The mean pre- and post-operative ECC were 1839 ± 587 and 1618 ± 498 cells/mm^2^, respectively. The mean proportion of endothelial cell loss (ECL) was 11.46 ± 4.78%. The mean postoperative ACD was 3.05 ± 0.44 mm, which was detected by ultrasound biomicroscopy (UBM). Typical anterior segment and UBM images of the implanted PCIOL were exhibited in Fig. 3. The mean postoperative PCIOL tilt degree was 2.81 ± 1.41^°^, and the mean postoperative PCIOL decentration degree was 0.31 ± 0.13 mm, which was observed by UBM according to reported method [11].

**Table 2:** Functional results

Mean operative duration	36.62 ± 10.70 min
Mean LogMAR preoperative UDVA	1.25 ± 0.50
Mean LogMAR postoperative UDVA	0.41 ± 0.22^*^
Mean LogMAR preoperative BCVA	0.48 ± 0.25
Mean LogMAR postoperative BCVA	0.33 ± 0.24^*^
Mean preoperative IOP	13.42 ± 3.51 mmHg
Mean postoperative IOP	14.13 ± 3.97 mmHg^#^
Mean preoperative ECC	1839 ± 587 cells/mm^2^
Mean postoperative ECC	1618 ± 498 cells/mm^2^
Mean postoperative ECL percentage	11.46 ± 4.78%
Mean postoperativ ACD	3.05 ± 0.44 mm
Mean postoperativ PCIOL tilt	2.81 ± 1.41°
Mean postoperativ PCIOL decentration	0.31 ± 0.13 mm

^*^P < 0.01 vs. preoprative result

^#^*P* > 0.05 vs. preoprative result

*PCIOL* posterior chamber intraocular lens, *UDVA* uncorrected distance visual acuity, *BCVA* best corrected visual acuity, *IOP* intraocular pressure, *ECC* endothelial cell count, *ACD* anterior chamber depth, *ECL* endothelial cell loss, *LogMAR* logarithm of the minimum angle of resolution;

Table 3 lists the postoperative complications. Four patients with transient corneal edema (19.0%) and three patients with transiently elevated IOP (14.3%) were observed on the first day after operation. These complications were resolved within 1 week by topical medication. No obvious anterior chamber flare or inflammatory cells was observed. Other complications, such as pigment dispersion, vitreous hemorrhage, anterior chamber hemorrhage, CME, retinal detachment, suprachoroidal hemorrhage, reverse pupillary block, corneal endothelial decompensation or endophthalmitis, were not observed. No patient presented suture breakage, suture knot exposure, severe lens tilting or PCIOL decentration.

**Table 3:** Postoperative complications

Postoperative complications	*n* (%)
Transient corneal edema	4 (19.0)
Transient elevated IOP	3 (14.3)
Pigment dispersion	0
Vitreous hemorrhage	0
Anterior chamber hemorrhage	0
Cystoid macular edema	0
Retinal detachment	0
Suprachoroidal hemorrhage	0
Reverse pupillary block	0
Corneal endothelium decompensation	0
Endophthalmitis	0
Suture breakage	0
Suture knot exposure	0
PCIOL tilting > 5°	0
PCIOL decentration > 1 mm	0

*IOP* intraocular pressure, *PCIOL* posterior chamber intraocular lens

## Discussion

PCIOL fixated by scleral suture plays an important role in visual restoration of eyes without inadequate capsule support. In the present study, clinical observation results of a modified technique of transscleral suture fixation of PCIO were described. An improved visual outcome was demonstrated in our patients that the mean final postoperative UDVA was much better than preoperative UDVA after a mean follow-up period of 7.8 months. The complication rate was low, and no patient presented suture breakage, suture knot exposure, severe lens tilting or PCIOL decentration. No severe intraoperative or postoperative adverse events were found during the study period.

With respect to the surgical technique, there were several significant improvements in our technique as compared to the traditional transscleral suture fixation as follows: the treatment method of conjunctiva and sclera; the method of securing the haptic with the fixation suture; the number of points of PCIOL fixation; the choice of PCIOL; and the method of avoiding suture and knot erosion.

The treatment method of conjunctiva and sclera in our technique was improved from the method reported by Hoffman et al. [12]. Dissection of the scleral pocket initiated from a clear corneal incision may avoid the need for conjunctival dissection and scleral cautery. According to Hoffman’s method, the size of scleral pocket was 3 × 3 mm and the hollow needle was then inserted into the eye through a full-thickness passage of conjunctiva, scleral pocket roof, and scleral pocket bed. In our technique, the size of scleral pocket was smaller (only 3 × 2 mm), and the hollow needle was inserted into the eye through the pocket bed directly under the scooped-up scleral pocket roof. Therefore, conjunctiva, Tenon’s space, and scleral pocket roof maintain their native integrity, which may not only increase postoperative comfort of patients, but also be helpful for the future glaucoma infiltration surgery [4, 13].

To avoid exposure of the suture knots, making triangular scleral flap to cover the knots is one of the most common techniques [4]. In order to make triangular scleral flap, conjunctival dissection, triangular scleral incision, lamellar scleral separation and hemostasis are needed, and such process is time consuming and cannot avoid bleeding. Our technique used the scleral pocket to omit the above steps. Therefore, the operative duration was greatly shortened by simplifying the surgical procedures, leading to few bleeding in the surgical field. The average operative duration of our patients was 36.62 ± 10.70 min, which might also reduce the incidence of intraoperative complications and shorten the postoperative recovery time. Moreover, compared with scleral flaps, which may tend to atrophy with time due to the small flap [12], the scleral pocket bed also provided a larger area for needle and suture passing [12], and the roof of the scleral pocket offered a greater surface area to cover the knots and suture ends.

Suture degradation can induce PCIOL dislocation in sutured scleral-fixated PCIOL [9], and breakage of 10–0 polypropylene sutures may happen after 3 to 8 years [3]. Therefore, we used 8–0 polypropylene suture, which was less biodegradable than 9–0 or 10–0 polypropylene suture. Subsequent progressive loosening of the knot tying on PCIOL haptic can also induce knot slippage [9]. For our technique, the suture was tied on the haptic by cow-hitch knot with no free suture ends, which could prevent knot loosening. It could also reduce the potential risk of uveitis induced by intraocular free suture ends chafing iris or ciliary body [10].

The positioning of the PCIOL is important for the long-term visual outcome. It has been reported that tilt greater than 15° causes coma aberration that cannot be corrected with spectacles [10]. The 2-point fixation technique using PCIOL with large C-shaped haptics is the most common technique [7]. However, the PCIOL tilt reported in 2-point fixation techniques can result in significant visual disturbances, including uncorrectable coma aberration, myopic shift and oblique astigmatism [14]. For 2-point fixation technique, the two suture-haptic junction points constitute and act as an axis to cause PCIOL tilting along. Subsequently, 3- and 4-point fixation techniques have been described [14, 15]. Recently, sutureless technique for PCIOLs implantation is becoming popular, and this method is also essentially a kind of 2-point fixation [9]. We adopted a 6-point suture fixation technique to prevent PCIOL tilting, as the fixation points of the three scleral pockets could constitute a stable plane rather than a rotating axis. Our technique selected the fixation points at 4, 8 and 12 o’clock to form an equilateral triangular fixation to counteract the torque and to avoid the ciliary artery. The mean PCIOL tilt degree in our patients was 2.81 ± 1.41°. It’s important that the fixation number must be balanced against the greater risk of complications from multiple suture passes through the sclera, uvea and vitreous cavity [7]. No suture passing-related complications, such as vitreous hemorrhage or anterior chamber hemorrhage, were found during our surgeries.

Optical decentration is a known complication of transscleral suture fixation of PCIOLs, inducing lateral shift of focus and radial astigmatism [14]. It has been reported that decentration greater than 1.0 mm causes radial astigmatism [10]. In addition, overtightening of fixation suture can cause greater anterior shift of the PCIOL optic than when the fixation suture is properly tightened. This anterior shift might work synergistically with the PCIOL optic and has a greater effect on pupillary capture of the PCIOL [16]. Therefore, symmetric fixation of a PCIOL will help prevent PCIOL pupillary capture [11]. Our fixation technique adjusted the suture tension in the operation and made the torque to be balanced, and the exact PCIOL center position could be observed and located through surgical field. The mean PCIOL decentration degree of our technology was 0.31 ± 0.13 mm. No pupillary capture was detected in our patients.

Zeiss CT ASPHINA 603P PCIOL was suitable for our technology. Firstly, it was a foldable PCIOL which could be inserted through a 3~3.5-mm corneal incision, leading to reduced final corneal astigmatism. Secondly, it had 3 close-looped haptics 120 ° apart which was just corresponding to the fixated position. Thirdly, its close-looped haptic was almost non-deformable, tear resistant and easy for the tying of the girth hitch. This haptic can not only prevent slippaging of knot along the haptic, but also prevent continuous cutting force induced by monofilament suture across the haptic [17]. Fourthly, the haptic loop was 10 degree backward angled with the optic, and then the optic was kept away from the iris to avoid chafing of the iris or ciliary body after suture fixation [18]. No pigment dispersion or glaucoma was found in our patients during their follow-up period, although pigment dispersion and pigmentary glaucoma are more prone to those undergo transscleral suture fixation of PCIOLs [19].

Although no severe postoperative complications, including PCIOL titling and decentration, were found in our modified technique during the follow-up period, the long-term endothelial safety, the posterior iris surface safety, and the stability of PCIOL position still need to be observed.

## Conclusions

Collectively, the technique of transscleral suture fixation of a foldable 3-looped haptics one-piece PCIOL through scleral pockets was a feasible method of PCIOL implantation in eyes with inadequate capsular support, which improved anatomic and visual outcomes with a low incidence of complications.

## Abbreviations

ACD: Anterior chamber depth
BCVA: Best corrected visual acuity
CME: cystoid macular edema
ECC: Endothelial cell count
ECL: Endothelial cell loss
IOP: Intraocular pressure
LogMAR: Logarithm of the minimum angle of resolution
OVD: Ophthalmic viscosurgical device
PCIOL: Posterior chamber intraocular lens;
UBM: Ultrasound biomicroscope
UDVA: Uncorrected distance visual acuity
